# Mental Nurses in Military Hospitals

**Published:** 1916-08-26

**Authors:** 


### THE MATRONS' AND SISTERS' DEPARTMENT.

### MENTAL NURSES IN MILITARY HOSPITALS.

In another column (p. 495) we print a letter from a qualified mental nurse in possession of the certificate of the Medico-Psychological Association, urging the claims of the asylum nurse to be admitted into military hospitals on the same footing as trained general nurses. No doubt there is something to be said in favour of her plea, and a few such nurses might perhaps be employed in those military hospitals in which there are patients affected with mental disease; but our correspondent would ridicule the notion that a nurse who had had only a general training in medicine and surgery would therefore be competent to nurse patients with mental disease, and she must make up her mind that a nurse who had been thoroughly trained in medicine and surgery would equally ridicule the notion that an asylum-trained nurse would be competent to take charge of medical, and especially of surgical, cases. It is quite true that a patient with a cut throat is occasionally admitted into an asylum, and that minor injuries, and sometimes even major injuries, occur in asylums as they do everywhere else; but in spite of our correspondent's assurance, we cannot admit that patients with cut throats pour into an asylum in appreciable numbers. We doubt if one asylum in fifty receives a single cut throat in a year, and when our correspondent says that a probationer in her first three months can dress wounds, she only shows that a qualified mental nurse in possession of the certificate of the Medico-Psychological Association can be completely ignorant of surgical nursing. Our correspondent asks what of epileptics, often admitted scalded from head to foot, or severely burned? The reply is that such cases are practically never seen in asylums. No asylum authority would admit that such a case ever occurs in an asylum, and if it were to occur outside, it would be taken, not to an asylum, but to a general hospital. It is farcical to suppose that surgical nursing can be learnt thus.

That a nurse who has had a three-years' training in an asylum would do better in a general hospital than a V.A.D. who has had no hospital training at all may be admitted. The first would, at any rate, be familiar with ward work and ward discipline, but in technical surgical training there would be little to choose between them. The V.A.D. has this advantage, however, that she can scarcely fail to know how ignorant she is. The mental nurse, while just as ignorant, does not know it.

The certificate of the Medico-Psychological Association is a guarantee that the holder of it is sufficiently trained in the nursing of mental cases, but it does not certify that the holder is a competent medical or surgical nurse, and, if it did, it would say what is not true. In the old days, before the Conjoint Board was formed, the M.R.C.S. was legally a diploma in medicine, but the medical examination was a farce, and the diploma was no real guarantee that its holder had any knowledge of medicine. It is the same now with the certificate of the Medico-Psychological Association. It is a diploma, not in nursing generally, but in a special department of nursing, and outside of that department it is of no value. Whatever smattering of surgical nursing a holder of this certificate has is acquired exclusively from books, and, incidentally, we may say that book-work has much too large a share in the preparation for this examination.

Another disadvantage under which nurses often lie who specialise in mental diseases is that they are, in comparison with medical and surgical nurses, of an inferior social class, and do not mingle comfortably with their colleagues in other departments. Attempts, which have been fairly successful, have been made in certain asylums to employ ladies as nurses, but in nearly all asylums the female nurses are of the servant class. Many of them are excellent women, and excellent mental nurses, conscientious, capable, assiduous, and devoted, as is our correspondent, no doubt; but, after all, a mental nurse is not a surgical nurse, any more than a surgical nurse is a mental nurse, and to put either in charge of cases that she has not been trained for is to invite disaster. Facts are facts, and are proverbially stubborn. It may or may not be unreasonable for a refined and educated lady, of good birth and breeding, to object to be placed under the orders of a woman of the class that she is accustomed to see waiting at her table; it may or may not be unreasonable of her to object to being associated in her work with a woman of that class, but, reasonable or unreasonable, the objection exists and is felt. Mental nurses cannot expect to be admitted to an equality with other nurses as long as they are drawn from a different social class; and they must not expect to be permitted to nurse surgical cases until they have had a thorough training in surgical nursing, a training that is not to be had in any asylum.

